# Barriers and beliefs: a comparative case study of how university educators understand the datafication of higher education systems

**DOI:** 10.1186/s41239-023-00402-9

**Published:** 2023-06-12

**Authors:** Bonnie Stewart, Erica Miklas, Samantha Szcyrek, Thu Le

**Affiliations:** Faculty of Education, University of Windsor, 401 Sunset Ave, Windsor, ON Canada

**Keywords:** Datafication, Higher education, Data, Digital education, Data ethics

## Abstract

In recent decades, higher education institutions around the world have come to depend on complex digital infrastructures. In addition to registration, financial, and other operations platforms, digital classroom tools with built-in learning analytics capacities underpin many course delivery options. Taken together, these intersecting digital systems collect vast amounts of data from students, staff, and faculty. Educators’ work environments—and knowledge about their work environments—have been shifted by this rise in pervasive datafication. In this paper, we overview the ways faculty in a variety of institutional status positions and geographic locales understand this shift and make sense of the datafied infrastructures of their institutions. We present findings from a comparative case study (CCS) of university educators in six countries, examining participants’ knowledge, practices, experiences, and perspectives in relation to datafication, while tracing patterns across contexts. We draw on individual, systemic, and historical axes of comparison to demonstrate that in spite of structural barriers to educator data literacy, professionals teaching in higher education do have strong and informed ethical and pedagogical perspectives on datafication that warrant greater attention. Our study suggests a distinction between the understandings educators have of data processes, or technical specifics of datafication on campuses, and their understanding of big picture data paradigms and ethical implications. Educators were found to be far more knowledgeable and comfortable in paradigm discussions than they were in process ones, partly due to structural barriers that limit their involvement at the process level.

**Graphical Abstract:**

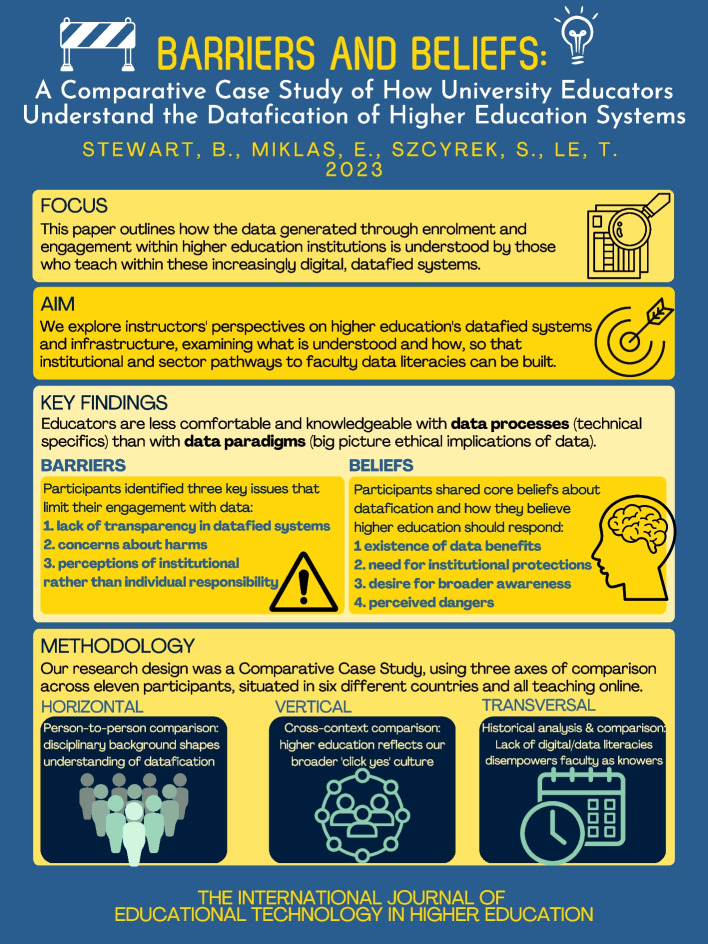

## Introduction

Over the past decade, digital datafied systems have become pervasive in contemporary life and higher education. Digital classroom platforms combine with tools for research, registration, communications, credentialing, finances, and other academic purposes to inform institutional decision-making and operations (Lane & Finsel, 2014). Procured via corporate entities and brokers (Decuypere & Williamson, 2021), these various digital tools comprise a complex, piecemeal infrastructure by which the operations of academic institutions are increasingly governed (Williamson, 2015). They are designed to extract machine-readable data at a scale not manually conceivable, and may monitor and track log ins, searches, keystrokes (Mozur et al, 2022), and even deleted text (Cyphers & Gullo, 2020). Yet the processes by which digital data is generated, communicated, and represented are seldom transparent (Atenas et al., 2020). Data extraction tends to remain relatively invisible to users even as personal information is algorithmically filtered and funneled into metrics that inform institutional decision-making (Szcyrek & Stewart, 2022).

The implications of this mass adoption of tracking and surveillance capacity for pedagogy, for academic governance, and for student—and teacher—data privacy represent a new professional landscape for many higher education instructors. In academia, models for how the world works are posited, researched, tested, and contested. Yet, as Google Research Director Peter Norvig declared in 2008, in a world deluged by what was then becoming known as Big Data, “all models are wrong, and you can succeed without them” (Anderson, 2008). The impact of this negation of theory for vast assemblies of often-decontextualized information is one that higher education has yet to fully grapple with. The academic tradition of the scientific method relies on the principle that correlation does not equal causation, but the statistical associations that datafication enables at scale are correlational, based on identifiable patterns rather than explainers. Datafication, therefore, represents a shift in both processes and paradigm for higher education.

The dominant discourse on datafication in higher education has been technical, focused on optimization of datafied processes. Focus is on utilizing data and learning analytics to personalize learning (Baker, 2016) or to support and improve student retention and success (de Freitas et al, 2014; Siemens, 2013). However, a critical discourse surrounding data has also emerged (Dalton & Thatcher, 2014; Raffaghelli et al., 2020). It differs from the technical approach in framing the ideas of objective data and optimization as specific positions grounded in particular assumptions, often reinforcing status quo power relations.

Some of the critical discourse has focused on race and gender biases (Benjamin, 2019; Noble, 2018) andclass discrimination (Eubanks, 2017) built into algorithmic decision-making processes. Data-driven systems do not just reflect the racism, sexism, and other oppressions of human societies, but participate in reinforcing them (D'Ignazio & Klein, 2020). The algorithmic logics that govern datafied decision-making have been shown to reinforce historical forms of colonization (Ricaurte, 2019) as well as opaque and discriminatory practices. This can lead to the ‘digital redlining’ (Gillard & Culik, 2016) of differential institutional access to digital services, or to students being refused funding for education if lending models deem their zip codes risky (O'Neil, 2016).

Critical approaches to datafication have surfaced additional, intersecting risks posed by datafied classroom platforms and broader systems. These risks include hacking of data, potential market exploitation of student data, reinforcement of knowledge monopolies in academic publishing (Lamdan, 2022), pedagogical overemphasis on what is countable (Williamson et al., 2020), and stigmatization of students via cross-campus data sharing (Marachi & Quill, 2020). Issues of privacy, surveillance, discrimination, and lack of consent became particularly visible in debates over online proctoring and exam tools during the emergency remote teaching (ERT) period of the COVID-19 pandemic (Swauger, 2020; Tufekci, 2020).

More broadly, automated machine learning and analytics tools tend to promise ‘personalization’ and insights into the relationships between teaching methods and student performance (Wong & Li, 2020). Yet even early in Learning Analytics (LA) literature, cautions and risks emerge, particularly with relation to ethics and limitations. Siemens (2013) noted that legal protections and regulations were “immature in relation to privacy and ethics concerns” (p. 1380). MacCarthy’s (2014) overview of predictive analytics-based interventions found they did reduce drop-out rates somewhat, but also bypassed or even undermined privacy norms by gathering masses of information not covered by consent. Bozkurt and Sharma (2022) analyzed LA research and found data-dominated educational landscapes that neglect the social and ethical aspects of teaching and learning in digital environments.

In spite of these noted concerns, datafication of higher education operations has increased apace. Yet concurrent approaches to data literacies—among faculty and students—have focused primarily on technical or process skills, with minimal emphasis on critical, ethical, or paradigm issues (Raffaghelli & Stewart, 2020), or on data governance. Institutional conversations about datafied tools tend to be siloed, with Information Technology (IT) experts often at separate tables from decision-makers or educators.

These silos reinforce and naturalize broader cultural norms. Draper and Turow (2019) coined the phenomenon of ‘digital resignation,’ wherein feelings of futility and helplessness in relation to digital surveillance are generated through routine corporate practices and uneven power relationships between corporations and publics. The contemporary digital landscape also conditions users to click ‘yes’ or ‘agree’ for access to the intended use of a tool (Newitz, 2005), waiving privacy and security rights for access, even to essential services. End User License Agreements (EULAs) confront users with Terms of Service (TOS) and Privacy Statements written in legal jargon, binding users to policies and agreements that they often cannot read or understand (Robinson & Zhu, 2020). As Sadowski (2019) notes, EULAs are one-sided forms of compliance rather than informed consent, and they pressure users towards acceptance of limited actionable choices. This complex reality is the cultural backdrop of our study, with higher education as both its site and engine for investigation. While we recognize that the issue of datafication affects all academic stakeholders, we focus on educators, who have traditionally been knowers within the academic environment and sources of knowledge for students.

## Study aims

This study developed out of an international 2020 pilot survey (Stewart & Lyons, 2021) of educators teaching online at universities, at the peak of the COVID-19 online pivot. That pilot survey explored faculty understandings of datafication through four proxy questions examining knowledge, practices, perspectives, and experiences regarding datafied systems being used in their institutions at that time. The findings of the survey suggested that educators have deeply-held beliefs what universities should be doing in relation to data and ethics, but indicated that these beliefs do not translate into high levels of knowledge or cautionary practice surrounding data and classroom tools. This gap between what representatives of the academy are doing—or are knowledgeable enough to be able to do—and what they believe their institutions should do in relation to datafication led to this follow-up qualitative study.

This case study expands on the pilot survey’s four proxy questions of knowledge, practices, perspectives, and experiences. It focuses on assessing how faculty members in various university teaching positions in various countries think about datafication, risk, and the classroom tools they used in their pandemic-era teaching, and aims to inform both policy and professional development in higher education.

## Methods

### Research design

Our team chose comparative case study (CCS) method for our research design. In CCS, a case can be a single entity, such as an individual, a small group, an organization, or a partnership (Creswell & Poth, 2018), a collective entity, such as a community, relationship, a decision process, (Yin, 2014), or an instance or data point, such as a survey respondent or subject in an experiment (Kaarbo & Beasley, 1999; King et al., 1994). In our study, each individual participant was considered both a case—a university educator with unique experiences and geopolitical and institutional contexts—and part of the larger collective case of university educators teaching online during the COVID-19 pandemic. CCS allowed us to examine cases as specific units of analysis, comparing and contrasting participants as individuals, but also tracing patterns and commonalities across participants as representatives of a specific historical context (Bartlett & Vavrus, 2017).

The CCS approach “promotes a model of multi-sited fieldwork that studies through and across sites and scales” (Bartlett & Vavrus, 2017, p. 15). CCS encourages focus on horizontal, vertical, and transversal elements of comparison. The horizontal dimension of CCS focuses on analyzing and tracing differences and similarities between participants, while the vertical dimension traces phenomena across scale, as socially-produced influences that impact and shape individual cases. Finally, the transversal dimension of CCS situates analysis historically, in specific power relations of time and place.

### Participants

Participants for the case study were recruited from the team’s 2020 pilot survey, which had 339 participants from 25 countries. Over 60 respondents from 10 countries agreed to potentially be contacted for a future follow-up. When our follow-up case study was funded and cleared through our institution’s Research Ethics Board, our team identified 20 potential volunteers from the survey to whom we reached out by email. We sought 10–14 participants for our case study. As we wanted to ensure a broad collection of individual cases, we initially invited potential participants from 10 countries and a variety of higher education teaching roles. 12 potential participants from six countries replied. No full professors consented to participate, and our final sample included fewer precarious or adjunct instructors than we would have liked, given the growing casualization of higher education teaching (Clark, 2019; Gupta et al, 2016).

Our study ultimately was comprised of 11 participants based in six countries: Canada, the United States, Mexico, Saudi Arabia, Ireland, and Scotland. We had eight women participants, from a variety of roles: one Associate Professor, two Assistant Professors, one Lecturer, one Learning Technologist, one Coordinator of Teaching and Learning, one Program Coordinator teaching within her own program, and one Adjunct Professor. The women taught in faculties of Law, Arts, Science, Nursing, and Education, as well as in faculty development roles. Three men participated in the study also. All three were Associate Professors, teaching Business, Engineering, and Computer Science. The 11 participants were all teaching online or anticipating teaching online at the time of the 2020 pilot survey, and most were still teaching online during the 2021 qualitative study. The majority had begun teaching online either in the year leading up to the pandemic or during the pandemic, though a couple had more longstanding online expertise. All participant interviews were conducted in English.

### Procedure and analysis

The study utilized semi-structured interviews as a primary data collection method, with participant field notes added to enable deeper insight into experiences and comparison between and across cases.

Semi-structured online interviews were scheduled with each of the 11 participants, and conducted over Zoom. Interview questions were based on the four proxy categories from the 2020 survey—knowledge, perspectives, practices, and experiences—and were organized into three sections. The first section focused on institutional contexts as influences on faculty knowledge and experience in relation to online teaching, exploring how educators learn about the platforms they teach on, and the directives and training available. The second section explored individual knowledge, experiences, and practices related to datafication, and how these were developed. It examined what faculty know about what happens to the data generated by use of their teaching platforms, and how they learn about data issues and practices. It also asked about perceived gaps in their knowledge, experiences with data breaches, and other risks, exploring how experiences have shaped existing perspectives and practices. The third section examined the perspectives of educators, posing questions about the benefits and risks they think datafication may pose for students or faculty, as well as for the role of higher education in society. It asked what they think should happen with classroom data and whether they would support initiatives in the higher education sector to oversee or regulate data collection via classroom tools. Conversations were encouraged to emerge and diverge from the core interview script.

Each interview took approximately 55–75 min. Interviews were recorded on Zoom, transcribed using automated transcription tool Otter AI, and—due to limited legibility of transcripts—re-transcribed in key areas by hand, by members of the research team. Transcripts were sent back to participants for verification.

Participants were also invited at the time of confirming their consent to collect and submit field notes, or brief written accounts of instances where datafication in their higher education digital classroom spaces became visible. The field notes were to be collected over a 6 week period during the online term, and could include screenshots of institutional or platform notifications in addition to written notes. The field notes questions were similar to those asked in the interviews, but were meant to explore specifics that might be lost or generalized in an interview context. Five of 11 participants submitted brief field notes, which were then integrated with the interview transcripts and analyzed as part of the overall project data.

To code and analyze data, our team used Braun and Clarke’s (2006) reflexive version of thematic analysis, which focuses on being deliberate, thoughtful, and reflective. The five-stage analysis process includes data familiarization, coding, thematic extraction, reviewing themes, and naming themes (Braun & Clarke, 2022).

In the data familiarization process, our team read through the automatically generated transcripts and corrected them based on recordings, where needed. Transcripts were sent back to participants for approval or further clarification. Because our intention was for the study to be open and participatory, we understood rigour as commitment to credibility and confirmability to participants (Guba & Lincoln, 2005) in the eyes of participants and the community of scholars to which they belong. Participants also chose their own pseudonyms for the results section.

In coding, we pulled out relevant quotes and excerpts that mapped to the 2020 survey proxy categories, which were highlighted and coded in Dedoose qualitative software to trace commonalities and distinctions. 95 codes were created. Then, in the thematic extraction process, each of the codes and how they might connect to larger themes were discussed. The team then combined and merged codes, using mindmaps, to formulate the first draft themes. This resulted in 10 potential sub-themes which were then merged into two overall themes of beliefs and barriers, each with sub-themes. After the overall themes had been named, the codes and themes were re-organized and analyzed according to the three core elements of CCS: horizontal or person-to-person analysis, vertical or person to structure (and structure to policy) analysis, and transversal or societal level analysis.

## Results and participant voices

**Fig. 1:**
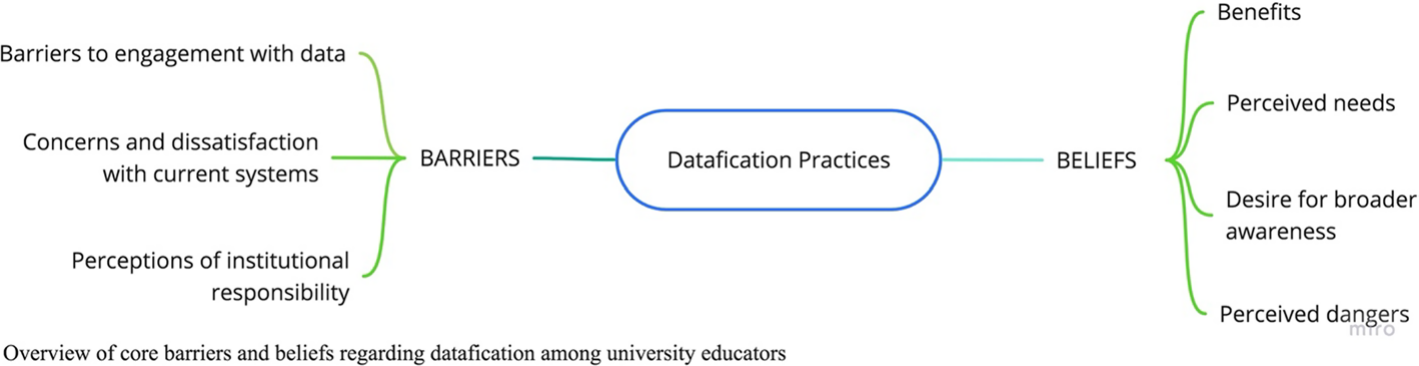
Overview of core barriers and beliefs regarding datafication among university educators

The first core theme to emerge from our analysis of the transcripts and mindmaps was barriers, or structural challenges within higher education that limit faculty understanding and engagement with the datafied systems that govern contemporary academic work. In the 2020 survey, to which all participants contributed, it was clear that faculty knowledge and practices were limited in relation to the proxy questions (Stewart & Lyons, 2021). In this follow up case study, we delved deeper into why, and identified three sub-themes: barriers to engagement with data, concerns and dissatisfaction with current systems, and perceptions of institutional responsibility (Fig. 1).

The second major theme that emerged from the case study was beliefs, encompassing participants’ perceptions and understanding of their experiences with the datafication of higher education. Again, in the pilot survey, it had been clear that participants’ perspectives on datafication were far broader than their specific knowledge, and our team wanted to understand why while also inquiring more deeply into those perspectives. Under the theme of beliefs, we identified four sub-themes: benefits, perceived needs, desire for broader awareness, and perceived dangers.

### Theme 1: barriers

Our first theme was barriers to engagement, as participants indicated that datafied systems are not generally transparent to educators using them. Almost all participants were significantly more confident speaking about the general paradigm of datafication in higher education than they were about the specifics—or processes and practices—of data collected by their online classroom tools.

Denise: *As far as what the institution is doing with that data or whether they hold it... how many times do I comment to my students? How many times am I reading what they actually post? All of that could be datafied and made visible to someone. I mean, I certainly have tons of data on my students during the semester. I can look at all of their logs of their behavior in the course, but whether that's retained for them or me, I have no idea.*

Jangle: *Sadly, I don't think I know anything about that data. I don't know where it's going to go. And I should know that -- it's not to say that the university hasn't supplied that, but I don't recall seeing it. And I mean, partly I need to pay more attention to that.*

When asked whether they had read the TOS for the institutional platforms they were using, only one had. Most trusted that the institution had signed off on the platform and that overall, the risks that might exist were acceptable.

Ahmad: *For the most part, I'm assuming that our lawyers have probably written most of the crap that's on there so they understand what it says.*

Cardinal: *I have not investigated the university tools because I am choosing to believe that the university has.*

Susana: *If the employer wants me to then that's basically one (TOS) I won’t look up. I've already sold myself to them.*

One participant in faculty development noted that if educators don’t understand what the risks *are*, then they may think all comparable tools are equally risky.

Fiona: *I don't know if we...very clearly say why we’re using these tools…it is licensed, it is checked out. We do have to be careful because we have folks going off using Google Drive or Dropbox or Menti... they might not be transferring any sensitive data for all we know, but we would still have concerns.*

Accessibility barriers also contributed to lack of engagement with TOS and datafication disclaimers. Participants noted various reasons why reading TOS is not a part of their practice.

Claire: *I would say the length is a deterrent. The language used, it's not necessarily in plain language.*

Ahmad: *That's a bit of naive trust I guess, like, what could go wrong? What could go wrong with Zoom data? But in the moment of trying to get the service working and functioning, you just want to get it over with.*

Faculty who speak English as an additional language noted it can be particularly challenging to make sense of the legalese of TOS.

Ilse: *I'm not a native speaker of English. I speak certain kinds of English and I read certain kinds of English very well, but a lot of it is legal terms and I don't always know exactly what they mean... it's just a question of time and print that’s too small, and then very long paragraphs.*

However, the law professor in our study, a first-language English speaker, noted that even if you *can* read the legalese that marks TOS, their design functions as a barrier.

Kelly: *I might have more understanding than someone who hasn't gone to law school, but I'm not sure about that..*. *the reason I don't read them is because in my view, there's nothing you can do about them. Like you can't contest any of the terms. If you don't agree, you can't use the tool. So in many ways, it's futile to even bother reading them.*

There was also general sense that time was a barrier: participants were stretched and overwhelmed given the pandemic and their online teaching responsibilities, thus having data policy conversations was not their top priority.

Ilse: *Even though I'm interested and I care about this topic, I don't have time to keep up with it because there's too much else coming at you.*

The next barrier was dissatisfaction with the implications of pervasive, datafied systems, particularly in relation to automated decision-making and surveillance. There was significant concern expressed about student privacy, about bias, and about overfocus on data leading to the dehumanization of education, and these concerns served as barriers to educators engaging more with digital and datafied tools because they did not necessarily want to reinforce or contribute to those systems and their impacts.

Ahmad: *AI measurement of people online to determine whether or not we think they're cheating or not paying attention or whatever... I don't like that, and I don't see the value. It doesn't align with my own pedagogy in terms of control.*

Kelly: *In my mind, it's online surveillance, which will always be used to target particular individuals... faculty of color, Black faculty, and Indigenous faculty. The same with students and student groups.*

Ilse: *It makes us cog wheels in the machine. I feel if we're not careful, there are useful things to analytics, but it depends on what you do with those figures... what you measure is what becomes important. And I feel that we might be measuring the wrong thing, and that what's really important often cannot be measured*.

The third sub-theme under barriers was institutional responsibility. In addition to participants’ perceptions that responsibility for TOS and risk assessment should be institutional, we found that most participants received limited information about data policies or privacy from their institutions. Even when asked to collect data-related institutional communications over the course of a month or more via field notes, only two of 11 participants submitted direct institutional communications as part of those field notes. Most institutional communications around data were reported as transactional and compliance-focused.

Susana: I just vaguely remember that data was mentioned at some point, especially when they were looking at monitoring engagement. Then the question of who can flag a student and what information can we share and legitimate reasons under GDPR rules.

More than one participant noted gate-keeping of privacy information and institutional failure to engage conversations about data implications, even when asked directly.

Claire: *I know from my involvement with the Faculty Association...we were trying to get access to the Gmail contract* *and some review did occur... but then it got put on the back burner, partly from being* *stonewalled a little bit by administration in terms of responses to questions about that* *agreement. We have since moved to Outlook and have not seen anything...haven’t got any access or insight into, you know, what is being shared with those companies.*

Wayne: *There’s no discussion about the actual data collection implications on either platform. And I've actually asked the question, how can students and staff opt out of data collection practices in Canvas? But nobody answered me.*

Participants made clear that training and professional development around data and data policies is an institutional responsibility that is not being met. Additionally, institutions’ overall approach to digital tools as technical rather than pedagogical was framed as a barrier to effective online learning in general, and to faculty understandings of datafication.

Kelly: *Trainings are given by people who know a lot about the platform, but have not necessarily taught on (it). What's really helpful is to hear from faculty who might have taught a very similar course to yours, whether it's in seminar, large lecture, experiential, whatever it is… The technical assistants have lots of skills, but I'm just not sure any of those things were helpful in terms of designing curriculum and responding to student needs and really understanding the online platforms as teaching tools.*

Participants were clear that there are multiple barriers to educators being involved in datafication conversations, in their institutions and the sector overall.

### Theme 2: beliefs

Additionally, it was clear that educators generally have strong and specific beliefs about datafication in higher education. The beliefs shared centered both on classroom tools, and on the broader intersection of datafication with participants’ ideals about higher education.

First, in spite of challenges expressed, almost all participants articulated some benefits to online learning, at least in a pandemic context. In relation to digital classroom tools in particular, approximately a third of participants reported that they saw benefits in datafication, AI, and in learning analytics (LA) especially. These participants framed datafication as making their lives easier or eliminating biased approaches to online teaching. LA was also outlined as having benefits for students, in terms of prompts, and was highlighted as one of the benefits of digital learning. The potential for innovation was also noted. However, at least one participant noted that analytics collect data that may never be collated or used, institutionally.

Claire*: A benefit can be some of the auto-reminders for students, to help them. ‘Oh great, there’s that assignment. I need to get going on that.’ From an instructor perspective, getting some sort of notification if someone has disengaged or hasn’t accessed the platform. That’s helpful to me then to reach out to offer support...as opposed to sort of from a policing perspective.*

Jason: *I often wrestle with regulation. Mostly because when we regulate, we sort of say, none of it can happen, or this is the bar. But we don't really consider that bar in different contexts or in different areas and different things. And it becomes a disincentive, more often than not, to innovation. That's it. Simple. But if, if there's potential positive uses of AI, you've just kept that from happening.*

Denise: *You have to pull data to report to the federal and state governments and it has to match their official identification. But if the student says, I want to be called something else... that was made possible. And I felt like that was a huge benefit.*

Ahmad: *I look at analytics for personal reasons, to check the engagement... Institutionally, I do not think that they are being processed or being looked at.*

Not all participants shared the belief in benefits to datafication. The second sub-theme of perceived needs frames participants’ perspectives on how to navigate the sector’s shift towards datafied systems. Common perceived needs included a critical focus on the need for pedagogical understanding of digital and datafied tools, rather than solely a technological or optimization frame. Support for data ethics initiatives, for protecting institutional data from vendor capitalization, and for more discussion around datafication generally were also beliefs commonly expressed by participants.

Kelly: *I can't imagine a situation where it would benefit faculty, students or the institution to share private data and with a private corporation or a private entity. Because I think whenever you're relying on the positives of data online, you cannot rely on those positives without the corollary that there's a huge negative, which is surveillance and policing of faculty, students and staff.*

Fiona: *Data devalues what we're trying to do to some extent. It almost takes away from the human element. Like the gamification of these things so we're looking at percentage scores to improve accessibility, or this to improve that. At the end of the day, numbers are just numbers.*

Almost all educators in the study expressed beliefs about the need for institutions to protect student and faculty data, but recognized some of the complexity of that imperative. One noted that increasingly, digital platforms limit local control and autonomy of institutional systems, while another acknowledged that universities pay digital corporate providers a significant proportion of institutional funds and pay again with student and faculty data.

Susana: *The university used to have its own data servers, so we could store our research data or personnel files in a server that was in a room in the university. But now we've just moved everything to the Cloud, to Microsoft. So we lost that autonomy, we're now hooked to Microsoft. And the same with Canvas...we used to run a local instance on the university servers... last year, two years ago, it’s become centralized. Just having less dependence on just a few platforms can be important.*

Claire: *I think it would make sense to put some boundaries around what for-profit companies can do with data that universities essentially are paying them for.*

Participants highlighted the need for institutions across the higher education sector to band together to push back against for-profit use of academic data, and there was particular pushback against non-anonymized student or faculty data being visible to corporate platforms.

Cardinal: S*o if the higher ed sector said, ‘we’re not using your products unless you guarantee these things...’ I can imagine that theoretically being helpful in terms of purchasing power.*

Wayne: *It needs to be something where the vendor can't go ‘we don't need you’ individually to the universities... And it can't be just individual faculty calling out vendors. There needs to be a collective... And we need to be able to point to it because right now it's just piecemeal.*

Jason: *I don’t want non-anonymized data available to vendors. I'm not sure that that's in anybody's interest. There are better ways to seek funds than to sell data, especially when selling data can be very risky. Because we don't really know where that data actually goes.*

Desire for increased institutional and sector-level awareness and policy change related to data was a third sub-theme under the theme of beliefs. Generally, participants noted that data conversations were both limited and also ineffective at engaging faculty and students. Not quite half of the participants noted discussion around data and data ethics happening within their institution. Often they themselves were involved with or starting these conversations. Particularly, those involved in educational technologies or with digital professional networks were more willing to make changes and be involved, but also felt isolated in those conversations.

Ilse: *On campus in my department, I feel like I'm very often the only one shouting in the desert. And then people go, oh yeah, yeah. But then we're also busy with everything else.*

Wayne: *I don't think I have a voice. That's why sometimes I just I want to save my energy and not waste it trying to fix these problems internally because it's a lot of effort. Many of us on individual campuses who were trying to do it, it's hard to necessarily even get a seat at the table.*

Relatedly, participants wanted policy change, including if not limited to ideas like data ethics, but recognized that individuals could not make that institutional reality. There was focus on collective action, and on the broader context of contemporary corporate influences on academic governance.

Jangle: *You need a hub within the institution where it's their responsibility to read those. And you appoint people from a variety of different faculties who have a vested interest to say, ‘Here's your job to read through this and to let the rest of us know how it affects us.’ Both in terms of practice and ethics. I'm happy with that because practically I don't want to read that shit.*

Kelly:* I think it's useful for all of us to always remember that universities used to be run by academics... Not that there was technology back then like this, but they had control. They were looking at options from the lens of a professor, of an academic, of what's in the best interests of academic freedom. Not that that was perfect... but with our donor funding of the university, data mining and surveillance... what's at stake is academic freedom, what a university is.*

Four of eleven participants noted that they were aware of specific aspects of datafication because their job was within this realm of knowledge, but that it was not generally available within their institution. More expressed particular concerns not just for fellow educators but for students, and indicated a belief that higher education has a responsibility to teach students about data and privacy and to demand greater privacy possibilities from vendors.

Wayne: *Where are students in this conversation? Why are we not thinking about the students’ access to their own data that they've created in this system? If we're still holding the data, why can't students access their own data?*

Fiona: *I know it's very difficult on a technical level to facilitate if students do want to opt in or opt out, and obviously you're creating a bit more work, but I think on that classroom level they're just not informed. And I don't know if staff necessarily are that well-informed either. And it’s problematic in terms of their growth as students, we don't embed those digital skills, those critical skills.*

Jangle: *I hit agree to get what I want because otherwise I can't get into the system... these invasions of privacy, they are invasions. These conversations generally are for the benefit of the tech provider. I want to see them take much more of an active role in looking at the privacy of students, the privacy of faculty, the privacy of the people using these tools.*

Finally, the fourth sub-theme of belief that emerged was that participants believed there were dangers in the datafication process, and were wary of it overtaking their institutions, even if they were aware of few means to resisting it. Their most prominent concerns included AI bias, the reduction of what is important to what is countable, and what happens to user data.

Claire: *I think the algorithm is only as good as the people that create the algorithm.* *So if you've got particular biases built in, whether it's around race or disability or other equity, or minoritized groups or disadvantaged groups, the data is only as good as the assumptions behind the algorithm.*

Susana: *I'm really not sure the data should necessarily be collected in the first place … I mean I don't wanna be like ‘things were fine in the past’, but it's not necessary to collect this amount of data to make things function.*

A commonality within this subtheme was concern over education being reduced to what can be captured by datafied tools, as well as control of what matters in the classroom being outsourced to non-humans.

Jangle: *Data misses the nuances. It can't take in the nuances... you can tell by my tone of voice how I'm answering your questions. That's not going to come up in data...So there's a potential for miscommunication or for a lot of information to not be transferred. I think that what has been happening with the increase in the reliance on technology is data is the only thing that we look at. And that's a detriment.*

Jason: *But when we started looking at…AI measurement of people online to determine whether or not they're cheating or not paying attention or whatever, I don't like that and I don't see the value. It doesn't align with my own pedagogy in terms of control. Students in general have choices and they don't have to pay attention if they don't want to. I don't need it determined for me that they're not paying attention because in fact, that's not an AI concern... That should be my concern.*

At least three participants had specific concerns related to surveillance and its potential impacts on vulnerable populations, as well as broader concerns about the likelihood even for ethics or regulatory approaches to have differential impacts on different populations.

Susana: *I don't like the normalization for students, of surveillance...in the UK in particular, I'm thinking immigration and surveillance and the Home Office and the requirements that they have for attendance for international students. So I often worry about the interface between those two things. You know, when online engagement monitoring becomes immigration control.*

## Analysis and discussion

### Horizontal analysis

Analyzing case studies on the horizontal axis allows person-to-person comparisons between participants (Bartlett & Vavrus, 2017), demonstrating how similar phenomena may occur in distinct locations. In spite of participants holding very different academic teaching roles in 6 different countries across 8 different disciplines, our study traced many patterns of similarity. Participants consistently expressed concerns about datafication in a higher education context, and about student data being visible to corporate vendors. Participants shared specific concerns about AI and datafied tools enforcing racial bias in particular, though one also mentioned AI processes’ capacity to minimize human bias. Educators who were aware of students being harmed by proctoring tools or LMS privacy issues had particularly strong concerns about data sharing.

While five of the participants were based in Canada, there was no visible common perspective amongst Canadian participants that was absent from those from other geopolitical contexts. Nor did role appear to have a consistent impact on participants’ positionality in relation to datafication: both casual and fairly senior educators shared complex perspectives on data, with views ranging from naïve to critical, often in the same interview.

We noted from the outset that our participant set reflected a gender disparity within academia, wherein the three male participants had three of the four most secure roles (Assistant Professor) within the group of 11, and represented traditionally technical fields of Computer Science, Business, and Engineering. However, while disciplinary perspectives were reflected in participants’ viewpoints, and male participants were more likely to note benefits of datafication, both male and female participants overall had critical perspectives rather than purely ‘technical’ or optimization-focused views on datafication.

Horizontal comparison indicated that disciplinary background, however, shapes familiarity with the specific process elements of datafication. Four participants’ roles were technology-adjacent, in Computer Science, Film and Media, Digital Technologies, and faculty development/support, and these participants were more likely to have specialized knowledge and practices related to data. There was a significant difference in perspectives among those who were not professionally involved with technologies and datafication conversations, as they also tended to be more limited in their knowledge, more content using the analytics that their LMS provided, and more likely to mention datafication saving time or making instructors’ jobs easier.

This correlation between familiarity with data conversations and seemingly increased critical data literacies has implications for institutions as well as society at large. A misconception surrounding privacy and personal data is that users do not care to understand data privacy, when in fact users *are* interested and even willing to utilize manageable opportunities to protect data when structures allow (Ahvenainen, 2021). Those structures can be national as well as institutional. Europe's General Data Protection Regulation (GDPR) comprehensive privacy law (Klosowski, 2021), which governs two of our 11 participants’ nations, is more stringent than privacy laws governing countries that other participants work in. We did note that the two participants from GDPR nations were quite knowledgeable about datafication, particularly in terms of process details. However, we cannot attribute that to geography entirely as both also have disciplinary expertise and networks involved in datafication conversations.

As noted in the introduction to this paper, datafication manifests in higher education both as a set of technical processes and a paradigm shift. Faculty knowledge regarding data processes may be—often self-admittedly—low, but we discovered more in-depth perspectives around the paradigm element of datafication than have generally been acknowledged. Participants were wary of responsibility for data issues at the process level, which appeared to stem from a combination of digital resignation, lack of time, and structural barriers related to lack of transparency and clarity. But at the paradigm level, when educators were asked what *should* happen, they expressed strong pedagogical beliefs about student privacy and the purpose of higher education.

Horizontal comparison also indicated that institutionally, faculty in very different disciplinary and geographic contexts experience similar barriers at the paradigm level: they consistently reported being neither aware of nor invited into paradigm conversations that they were nonetheless fully capable of contributing to. We conclude that siloed data conversations based on process expertise, then, not only allow technical discourse around datafication to dominate institutional and sector discussions of discussion, they also tend to keep the paradigm conversation off the table entirely. Yet our study demonstrates that a broad range of educators are interested in the implications of big data for academic knowledge and for pedagogy, and have a great deal to contribute.

### Vertical analysis

The vertical axis of CCS recognizes that space is socially produced (Massey, 2005), and allows for analysis of the connections between individual and structure, and between structure and policy (Bartlett & Vavrus, 2017). At this level, we note further patterns related to discipline as well as to institutional and sector practices that fail to cultivate and recognize faculty knowledge in relation to datafication.

A majority of participants noted that digital TOS for classroom tools are too long and too complicated for non-specialists to read, yet acknowledged they agree to terms without reading them, because there are no real avenues to resistance. One participant highlighted that they had to click agree to their university’s COVID-19 protocol, which collected personal information, in order to gain access to campus. This is an example of a university producing and reproducing societal norms and spaces that protect neither faculty data nor faculty autonomy, but also fail to cultivate faculty understanding and capacity to resist data extraction.

We noted that technology-adjacent disciplines may produce—across disciplines—institutional spaces of data literacies development and critical expertise related to datafication, especially with regard to pedagogy and privacy. However, the study made clear that participants from these disciplines are not as involved in institutional decision-making regarding datafication as their expertise would indicate. In terms of social production of spaces for learning about datafication, institutions around the world appear not to be drawing on faculty expertise—even where it exists—to support and sustain critical and paradigm-level conversations about datafication.

Ultimately, vertical analysis indicates those conversations are lacking across the sector. Participants generally had limited institutional digital training, noting that their universities had not provided sufficient opportunities for them to engage pedagogically with digital tools or with issues of data. At least four participants expressed that if there were data conversations happening in their spaces, they were being left out. This points to the reality that limited faculty knowledge in relation to the paradigm and processes of datafication stems from a sector-wide failure to foster the social production of data literacies, for professionals and for students. That said, our data supports the premise that Europe may be doing slightly more than other jurisdictions, or at least those represented in our study. SHEILA, a European project focused on education, aims to engage critical stakeholders' participation in policy development in order to change institutional practices (SHEILA Project, n.d.). The presence of that kind of collective initiative can have an impact on national and institutional conversations, and our two European participants did appear in their interviews to have a broader sense of national higher education practice related to datafication than those in other geopolitical jurisdictions.

But ultimately, as higher education processes have become more specialized and datafied, inclusive, data-literacy-developing paradigm conversations have not been a primary focus in most institutions or geopolitical spaces, in spite of the educative role academia could serve on this front. Rather, IT issues, policy, and teaching appear to be, generally, treated separately. This creates a cycle we observed in our findings, where the barriers that educators face related to datafied systems create frustrations, wariness, and concerns, impacting trust in their institutions. Then, the beliefs about digital and datafied systems that emerge from those spaces of altered trust in turn reinforce barriers to engagement, as educators often do not believe that their involvement can or will make a difference.

### Transversal analysis

The transversal axis of CCS pertains to the historical, situated, contextual relations under investigation. Transversal analysis emphasizes how the globalizing process of datafication interconnects people and policies, including the impact that policies and practices have on people and their access to information (Bartlett & Vavrus, 2006; do Amaral, 2022).

This study offers a snapshot of higher education faculty grappling with a specific and global set of pressures—teaching online during the COVID-19 pandemic—while also navigating the historical arc of a decade of global higher education datafication. In addition to the noted paradigm shift in higher education’s epistemological underpinnings, the policies and practices that govern relations between educators and their institutions have also shifted in this period due to datafication. The rise of digital systems and extractive data collection practices has come to govern access to contemporary society. Higher education has neither been exempt from this process, nor—as our study demonstrates—taken an educative role in addressing it. This context produces subjects, even at the faculty level, who are simultaneously disengaged and disempowered as knowers due to forces largely beyond their personal control. Though the scope of our study does not include student perspectives, our participants were clear that current approaches to datafication do not build critical data literacies among higher education faculty, nor prepare them to do the same with students.

This study is not longitudinal and we have no comparisons with faculty from a different era. Still, the shift in how much educators know about the systems within which they work becomes visible through a transversal lens. Prior to this era, faculty generally worked and taught in institutional systems that they themselves had been subject to and trained in, and understood the implicit and explicit power relations governing both the four walls of the classroom and the academy more broadly. The rise of digital tools and datafication has altered those power relations and faculty’s power position as knowers within the academy. Our study indicates that educator still have significant knowledge and beliefs about the pedagogical implications of data, but suggests that the circulation of power and policy in this historical moment devalues faculty input and governance. It is concerning that, at this time of unprecedented digital and datafied dependence in higher education, even our most digitally adept faculty participants noted institutional barriers to their data literacies and their contributions to institutional decision-making surrounding data. Taken as a case together, as representatives of a specific instance in time, these educator participants make visible how the historic arc of datafication is one that—thus far—has put up barriers to full faculty participation in the changing landscape of academic governance.

## Conclusion

This paper explores how the data generated through enrolment and engagement within our institutions is understood by those of us who teach in contemporary higher education, and what we believe should happen from here. Our CCS method enables consideration of the data and the resulting themes through three methodological lenses, allowing us to present our findings not just as individual comparisons or representations of a profession, but as perspectives contextualized within institutional and societal contexts. Overall, the study demonstrates vividly that faculty work environments—and knowledge about their work environments—have been shifted by pervasive datafication. All participants appeared to grasp the pedagogical and ethical facets of that shift, even while experiencing barriers to knowledge and literacies in datafied processes. While a limitation of our study is its size, these findings were robustly reinforced across the variety of institutional status positions and geographic locales that the eleven participants came from.

Our study surfaced multiple institutional and structural barriers to educator data literacies and to shared governance on data issues. Lack of transparency and clarity were barriers to engagement with data, as were concerns and dissatisfaction with datafied systems, and perceptions of institutional rather than individual responsibility. Participants noted they have few institutional opportunities to engage in pedagogical and ethical conversations about data. However, the vast majority had some level of interest in the pedagogical implications of data, and—in spite of varied perceptions about the benefits of data within institutions—had strong beliefs about how the higher education sector can and should address the issues that data poses. Participants generally believed that institutions need to do more to protect student data as well as to protect racialized and minoritized students in particular. Educators expressed support for policy change related to data processes, especially in the context of privacy, and for increased awareness-raising and conversation about data paradigms and their implications. They also shared broad-ranging concerns about the dangers of datafication, particularly related to surveillance, outsourcing of classroom control, and undermining of the purpose and ethos of higher education.

Our hope is that by establishing what educators do know and think about data in higher education, this project can make visible the value of faculty perspectives in decision-making and direction-setting in relation to datafied systems. Overall, the study makes clear that in spite of data literacy barriers and the broader cultural phenomenon of digital resignation (Draper & Turow, 2019), university educators are invested in the ethical and pedagogical structures of academia. However, the ways conversations about data are structured on campuses tends to exclude their participation as knowers. The key takeaways from our comparative analysis amplify this critique of how datafication is addressed, as current practices focus on optimization and technical processes, and serve to shift power relations away from full faculty participation in academic governance. We encourage broad data ethics and data literacies initiatives across higher education as means to begin to address these impacts of datafication.

## Data Availability

The dataset analyzed in the current study is not publicly available due to potential identifiability of participants, but is available from the corresponding author upon reasonable request. Data from the 2020 pilot survey on which the case study was based, and which is referenced in the paper, is available as an open data set in the Zenodo repository: https://zenodo.org/record/4096183#.ZFrv9-zMLFo.
